# Gut microbiota — bidirectional modulator: role in inflammatory bowel disease and colorectal cancer

**DOI:** 10.3389/fimmu.2025.1523584

**Published:** 2025-04-30

**Authors:** Xilun Cui, Changfeng Li, Jing Zhong, Yuanda Liu, Pengtuo Xiao, Chang Liu, Mengwei Zhao, Wei Yang

**Affiliations:** ^1^ Department of Endoscopy Center, China-Japan Union Hospital of Jilin University, Changchun, China; ^2^ Department of Medical Imaging, The Third Affiliated Hospital of Changchun University of Chinese Medicine, Changchun, China; ^3^ Department of Immunology, College of Basic Medical Sciences, Jilin University, Changchun, China

**Keywords:** gut microbiota, CRC, IBD, immune modulation, SCFAs

## Abstract

The gut microbiota is a diverse ecosystem that significantly impacts human health and disease. This article focuses on how the gut microbiota interacts with inflammatory bowel diseases and colorectal tumors, especially through immune regulation. The gut microbiota plays a role in immune system development and regulation, while the body’s immune status can also affect the composition of the microbiota. These microorganisms exert pathogenic effects or correct disease states in gastrointestinal diseases through the actions of toxins and secretions, inhibition of immune responses, DNA damage, regulation of gene expression, and protein synthesis. The microbiota and its metabolites are essential in the development and progression of inflammatory bowel diseases and colorectal tumors. The complexity and bidirectionality of this connection with tumors and inflammation might render it a new therapeutic target. Hence, we explore therapeutic strategies for the gut microbiota, highlighting the potential of probiotics and fecal microbiota transplantation to restore or adjust the microbial community. Additionally, we address the challenges and future research directions in this area concerning inflammatory bowel diseases and colorectal tumors.

## Graphical Abstract

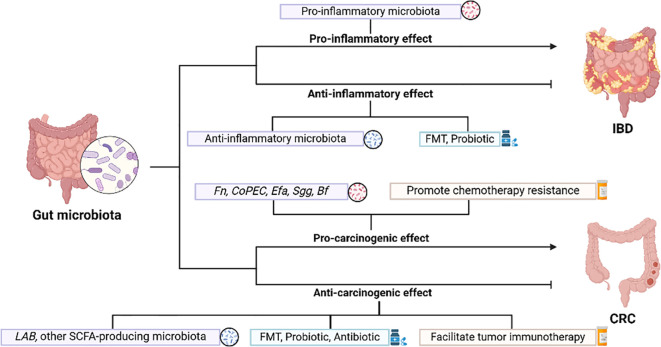

## 1 Introduction

The human body harbors a diverse range of microorganisms, encompassing bacteria, viruses, fungi, protozoa and so on, which make up about 1-3% of body weight. The intestinal microbiota is particularly rich, with about 1000 species, consisting mainly of bacteria (1). The number of intestinal microbiota is about equal to the number of human cells in the body, and they encode about 100 times more genes than the body’s own genes (2, 3). As the “second genome”, Intestinal microorganisms fulfill various functions, such as enhancing the host’s immune system, assisting in digestion, regulating intestinal hormone and nerve signals, influencing drug metabolism, detoxifying harmful substances, and affecting the production of metabolic compounds in the host (4). Increasing evidence shows that the gut microbiome directly influences immune regulation, supports the development of the immune system, and helps maintain its normal function. Conversely, the immune status of the body can also affect the composition of the gut microbiota. Thus, the microbiome plays a crucial role in both health and disease (5). Many studies have revealed a strong link between gut microbiota dysbiosis and gastrointestinal diseases, malignant tumors, cardiovascular diseases, neurological disorders, and other disorders in humans, of which tumors are one of the most studied diseases (6–12). The effect of gut microbes on immune function, including immunosuppression and activation of inflammation, has the most direct impact on GI diseases, the most prominent types of which are inflammation and neoplasms, because gut microbes colonize the intestinal tract.

Inflammatory bowel disease (IBD) is a gastrointestinal condition characterized by chronic inflammation, manifested by disorders of mucosal structure, altered intestinal microbial composition, and systemic biochemical abnormalities, and includes ulcerative colitis (UC) and Crohn’s disease (CD) (13). IBD can develop at any age, and is generally common in adolescents at the first onset of disease. Its incidence continues to rise globally, with a growing number of children and elderly individuals (14).The causes and pathogenesis of IBD remain not completely understood, and abnormalities in immunoregulation play a crucial role in the onset of IBD. Many immunosuppressants, biological agents and small molecule substances have been applied to the clinic and have achieved good efficacy in the treatment of IBD and its related complications. Patients with IBD face an increased risk of developing Colorectal cancer (CRC), called colitis-associated cancer, compared to normal subjects (15). Beneficial gut bacteria can exert immunosuppressive effects by modulating host immune cells, while harmful bacteria induce inflammatory cytokines through interactions with immune cells or their metabolites, leading to intestinal damage (16, 17). There are a number of bacteria that can act on both IBD and tumors together, such as a relative increase in *Escherichia coli* (*E. coli*) in the patient’s intestinal microbiota, and the abundance of *Bacteroides fragilis* (*Bf*) is strongly linked with both active IBD and CRC (18–20). With the substantial development of IBD genetic susceptibility and gut microecology research, future treatments may favor individual precision therapy by targeting the microbiota in order to improve patient symptoms and further enhance quality of life.

The long-term chronic inflammatory response in the intestine is associated with the occurrence of cancer. CRC is a common gastrointestinal tumor and the second leading cause of cancer-related deaths worldwide. Recent years have seen a rise in CRC incidence across various countries, and the incidence and mortality rates vary significantly around the world. According to the National Cancer Center of China, CRC ranks as the second most prevalent cancer in men and the fourth in women (21). As tumor immunology advances quickly, people have gained a more in-depth understanding of the body’s anti-tumor immune response pathway and the immune escape mechanism of tumors, and significant breakthroughs have been made in immunotherapy for tumors, but all of them have certain limitations. Most tumors are insensitive to the various existing immunotherapies, indicating that they have a strong immune tolerance mechanism. Coupled with the high degree of heterogeneity of tumors among patients, it is often difficult for existing single therapies to produce satisfactory results in clinical applications. Current metabolomics and macrogenomics research highlights the gastrointestinal microbiome’s dual role in preventing cancer, promoting tumor development, and influencing the effectiveness of anticancer treatments (22). Some bacteria in the gut microbiome exert pro-cancer effects, while others have tumor suppressor properties (23). Therefore, if the components of gut microbes can be artificially altered, they may not only serve as therapeutic targets, but also exert immunomodulatory functions.

Targeting gut microbes for the treatment of intestinal diseases has a promising future, and we summarize the current mechanisms of gut microbes in IBD and CRC, as well as the present state of targeting gut microbes for managing these diseases.

## 2 Gut microbiota and inflammatory bowel disease

IBD is a chronic, recurrent inflammatory disorder of the digestive tract linked to autoimmune processes. The composition and diversity of the intestinal microbiota are intimately linked to the onset and progression of IBD (24).

### 2.1 Anti-inflammatory effects of gut microbiota

**Figure 1:**
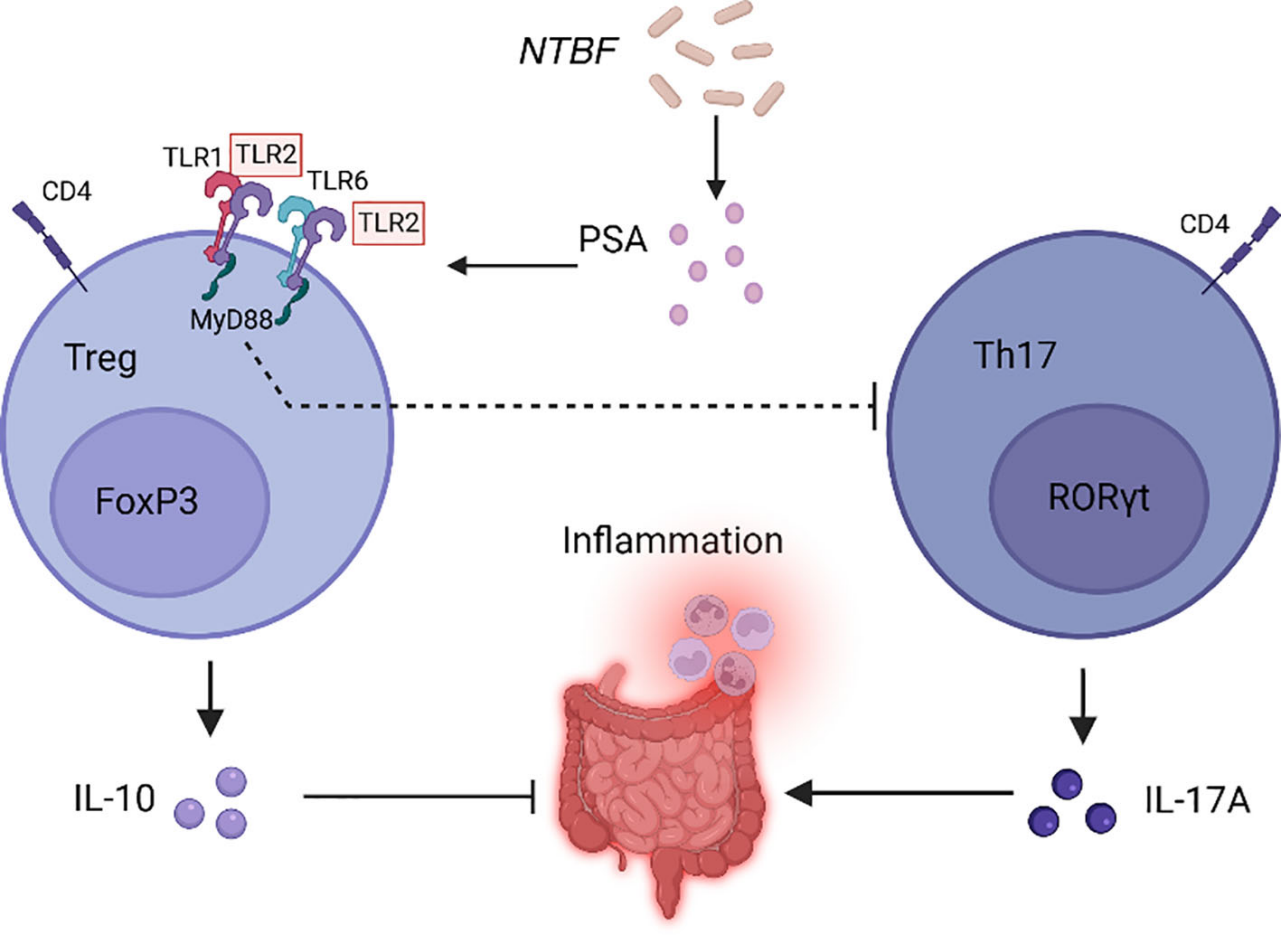
*NTBF* promotes immunologic tolerance. *NTBF* releases PSA through outer membrane vesicles, promoting the production of IL-10 through TLR2 signaling on CD4 Foxp3 Tregs, and inhibiting RORγt Th17, reducing the production of IL-17A, thereby promoting immune tolerance. (Foxp3, Forkhead Box P3, a key transcription factor for Treg, which is usually used as the marker for Treg. RORγt, retinoic acid related orphan receptor γt, a key transcription factor for the differentiation and function of Th17).

In the human gut microbiota, *Firmicutes* (*F*) and *Bacteroidetes* (*B*) constitute approximately 90% of the total bacterial population (25). IBD patients frequently demonstrate a decline in the *F*/*B* ratio, and studies have indicated that the diversity of *Firmicutes* in the intestinal microbiota of IBD individuals reduces, characterized by a significant decline in anaerobic bacteria of the class *Clostridia*, particularly the commensal bacterium *Faecalibacterium prausnitzii (F. prausnitzii)* (26–30). *F. prausnitzii* is capable of generating butyrate, which suppresses HDAC1, stimulates Foxp3, and blocks the downstream IL-6/STAT3/IL-17 pathway, maintaining the T helper 17 cell/regulatory T cell (Th17/Treg) balance and exerting substantial anti-inflammatory effects (31). Additionally, the abundance of *Phascolarctobacterium* is significantly decreased, and *Phascolarctobacterium*, when co-cultured with *Paraprevotella*, consumes succinate and produces the anti-inflammatory SCFA propionate (32, 33). The reduction of SCFA-producing *Phascolarctobacterium* in IBD patients implies that the anti-inflammatory impact of SCFA is potentially weakened, potentially exacerbating IBD symptoms (34). There are also some anti-inflammatory bacterial groups, such as *non-toxigenic Bacteroides fragilis* (*NTBF*), whose symbiotic factor polysaccharide A (PSA) can stimulate CD4+ T cells and induce the anti-inflammatory effects of Tregs through Toll-like receptor 2 (TLR2), and inhibiting Th17, promoting immune tolerance (**Figure 1**) (35). Tan et al. confirmed that new strains of *Bf HCK-B3* and *Bacteroides ovatus ELH-B2* can alleviate LPS-induced inflammation by regulating cytokine production or restoring the Treg/Th-17 balance (36).

### 2.2 Pro-inflammatory effects of gut microbiota

However, certain microbial groups can facilitate inflammatory responses in IBD. Adherent-invasive *E. coli* can induce epithelial mitochondrial fission, influencing intestinal permeability (37). *Enterococcus faecalis (Efa)* can enhance colonic cytokine expression and cause colitis (38). *ETBF* and its secreted zinc-dependent metalloprotease toxin, *B.fragilis* toxin (BFT), triggers Stat3 activation and Th17 immune responses, promoting mucosal permeability (39, 40). Additionally, Ha et al. discovered that *Clostridium innocuum* is abundant in the ‘creeping fat’ (CrF) of severe CD individuals, and this bacterial group can stimulate tissue remodeling, resulting in the formation of adipose tissue barriers (41). Henke et al. reported that Ruminococcus gnavus secretes a Toll-like receptor 4 (TLR4)-dependent glucorhamnan polysaccharide, effectively inducing dendritic cells to secrete inflammatory cytokines (TNF-α), thereby facilitating CD inflammatory responses (42).

**Figure 2:**
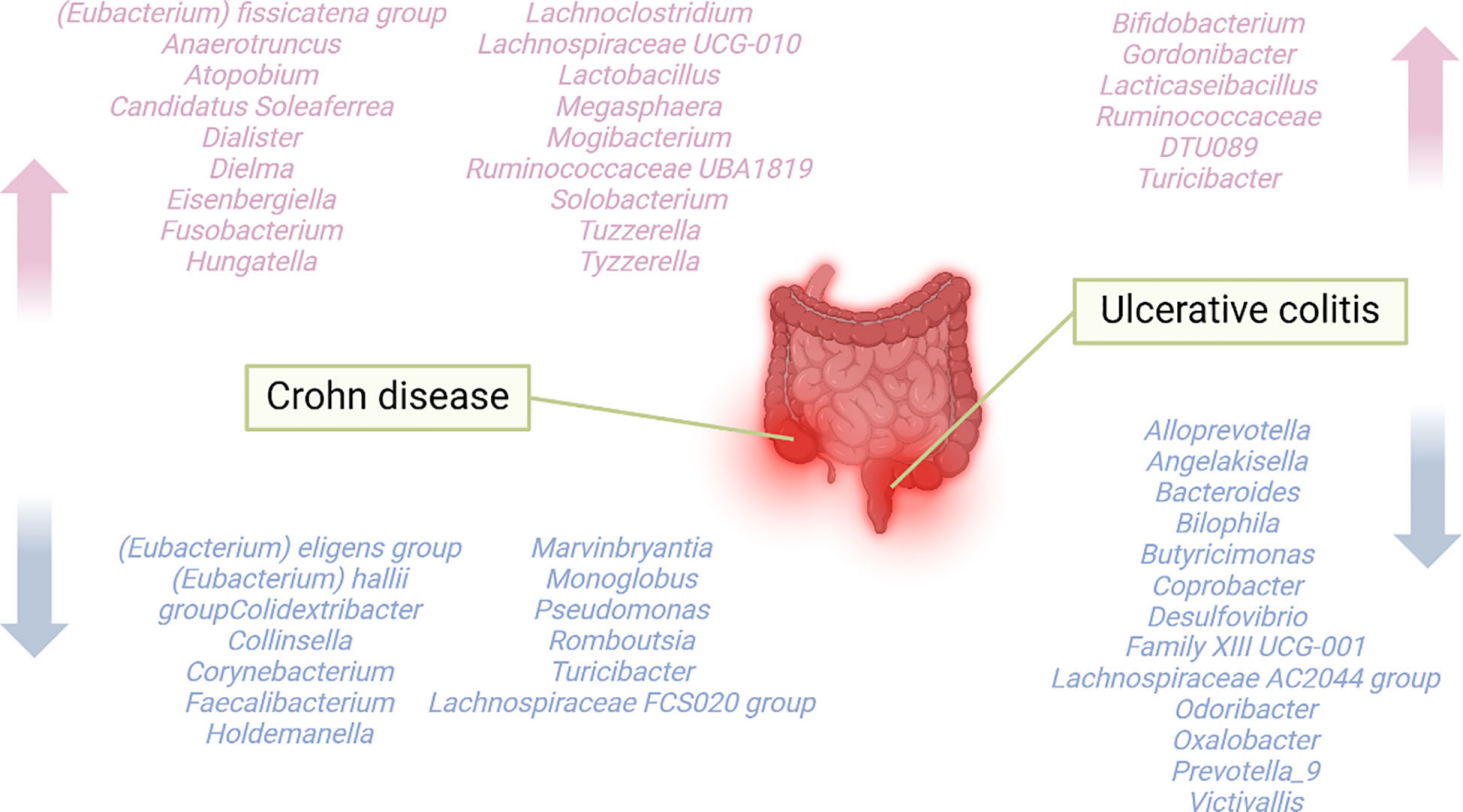
Overview of genera associated with CD or UC. To classify genera based only on their abundance being higher or lower in CD or UC compared to healthy individuals.

An investigation on gut microbiota characteristics revealed that within the dataset of genera related to IBD, the number of genera related to CD exceeds that related to UC (43). Additionally, in contrast to healthy individuals, the α-diversity of IBD is diminished, with a more pronounced reduction observed in CD; the microbial community composition (β-diversity) undergoes changes in UC, while the alterations in β-diversity are more conspicuous in CD (**Figure 2**) (43). Pascal et al. discovered that dysbiosis is notably more pronounced in CD individuals compared to those with UC, manifested as lower diversity and more unstable microbial communities (44). In CD patients, the levels of *Actinomyces*, *Veillonella*, and *E.coli* rose, while the enrichment of *Christensenellaceae*, *Coriobacteriaceae*, and particularly *Clostridium leptum* declined. Similarly, the level of *E.coli* also rose in UC patients, while the levels of *Eubacterium rectale* and *Akkermansia* decreased (45). *Intestinibacter* abundance increased in both CD and UC, whereas *Efa* abundance markedly decreased in CD (46).

### 2.3 Microbiome therapy in inflammatory bowel disease

Adjusting ecological dysbiosis, using fecal microbiota transplantation (FMT), and supplementing with probiotics, especially butyrate-producing bacteria, can help improve IBD (47). As a research hotspot, FMT therapy is capable of restoring the intestinal mucosal immune homeostasis in IBD patients. The first utilization of FMT in UC patients was documented in 1989 (48).

#### 2.3.1 FMT

FMT is suitable for moderate to severe IBD, particularly for active IBD accompanied by concurrent refractory or recurrent *Clostridium difficile* infection. Patients might demonstrate more prominent microbiota alterations and generate certain clinical effects. Studies have indicated that a single FMT is relatively safe (49, 50). Nevertheless, the alterations in the intestinal microbiota of the majority of patients are temporary. Chu et al.’s research implies that FMT is a complex mixture of biological entities interacting with the recipient’s microbiota and immune system. By monitoring the recipient’s microbiota, it was discovered that 10 weeks after receiving the treatment, the microbiota had significantly deviated from the donor bacteria (51). Hence, attention ought to be paid to the colonization dynamics of the gut microbiota during FMT treatment, and it is also requisite to conduct repetitive transplantation periodically to maintain the modified microbiota. Research has shown that multi-stage FMT can restore the recipient’s microbiota and induce remission in patients with active UC. This therapeutic approach leads to substantial alterations to the microbiota composition, encompassing an increase in the abundance of Firmicutes both during the FMT period and up to six months afterwards (52). Moayyedi et al. believe that intensified dosing and multi-donor FMT can effectively achieve both clinical and endoscopic remission in patients (53).

Genetic and physiological factors related to both the donor and the recipient will have an impact on the therapeutic effect. Liu et al. emphasized the significance of considering the activity of the gut microbiota in donor samples for FMT. A detailed analysis of the active microbiota is crucial for understanding the mechanisms behind the therapeutic effects of FMT (54). Angelberger et al. identified microbial types associated with disease severity and FMT treatment success, especially related to the enrichment of *Enterobacteriaceae* and the deficiency of *Lachnospiraceae (* 55). This subset of UC patients is more prone to be an effective subgroup for FMT treatment. Additionally, research has revealed that triple antibiotics (amoxicillin, fosfomycin, and metronidazole [AFM]) can synergize with FMT. Pretreatment with AFM to eliminate the recipient’s dysbiotic Bacteroidetes might facilitate the colonization of the donor’s Bacteroidetes bacteria during FMT, thereby alleviating the intestinal microbiota dysbiosis in UC patients caused by the loss of diversity among Bacteroidetes species (56). Regarding the short-term safety of FMT in IBD, multiple meta-analyses have shown no significant differences compared to the placebo group, with most adverse reactions being mild symptoms such as gastrointestinal discomfort and fever, and a low incidence of severe adverse events (57, 58). There is limited literature on long-term safety. One study mentioned that patients receiving FMT experienced certain adverse reactions in the long term, including rashes and myasthenia gravis (59). Additionally, another study reported that these patients had a comparable risk of developing severe diseases such as autoimmune disorders or tumors compared to the antibiotic group (60). In the future, further improvements in the preparation and delivery methods of fecal microbiota are needed to effectively manage these risks and enhance the safety and efficacy of FMT.

#### 2.3.2 Probiotic

VSL#3 is a probiotic mixture, and several clinical studies have indicated that it can effectively alleviate IBD symptoms (61, 62). VSL#3 encompasses eight types of bacteria, namely *Bifidobacterium breve*, *Bifidobacterium longum(B. longum)*, *Bifidobacterium infantis*, *Lactobacillus acidophilus*, *Lactobacillus plantarum*, *Lactobacillus paracasei*, *Lactobacillus delbrueckii*, and *Streptococcus thermophilus (* 63), which can lower the level of colonic mucosal inflammation by enhancing the intestinal environment, reshaping the microbial composition, and regulating specific bacterial levels, thereby treating or alleviating IBD symptoms (64, 65). Other literature reports that *Lactobacillus GG*, *B. longum*/Synergy 1, and *Bifidobacteria*-fermented milk can effectively ameliorate intestinal inflammation in UC individuals, with the role in maintaining remission (66).

*E. coli* Nissle 1917 (EcN) is the active strain of the microbial drug Mutaflor^®^, a non-pathogenic Gram-negative bacterium that is able to inhibit the growth of *Salmonella* and other pathogenic bacteria. It is the sole probiotic recommended in the ECCO guidelines for UC patients, and its efficacy in maintaining disease remission is comparable to that of mesalazine. The fimbriae F1C of EcN enables it to persistently colonize the intestinal epithelium and directly stimulate cells to produce defensins, preventing pathogens from adhering to and invading intestinal cells. It can also interact with the immune system, decreasing pro-inflammatory cytokines like IL-2, TNF-α, and IFN-γ, while elevating anti-inflammatory cytokines (67).

*Clostridium butyricum* (*CB*) is a probiotic utilized clinically for functional gastrointestinal disorders, and the butyrate it produces can ameliorate intestinal mucosal inflammation. Hayashi et al. reported that it can effectively induce colonic mucosal macrophages to generate the anti-inflammatory cytokine IL-10 through TLR2/MyD88 pathway, thereby suppressing intestinal inflammation in a mouse IBD model (68).

#### 2.3.3 Diagnostics and personalized medicine

The onset of IBD can be difficult to predict, and invasive examinations such as colonoscopy are currently commonly used to determine the inflammatory activity of IBD. There is growing interest in diagnosing, monitoring, and treating the disease non-invasively based on the gut microbiota. The rapid progress in synthetic biology offers the possibility of targeted microbial engineering, enabling engineered microorganisms to diagnose IBD by selectively detecting biomarkers. A study has designed an engineered probiotic, EcN, which non-invasively monitors disease activity in patients by detecting the IBD biomarker calprotectin in feces (69). Both nitrate and thiosulfate are biomarkers of intestinal inflammation. Woo et al. introduced a nitrate-responsive genetic circuit into EcN, enabling the biosensor to detect thiosulfate and nitrate, thereby aiding in the diagnosis of colitis (70). Zou et al. developed a smart responsive bacterium (i-ROBOT) composed of EcN, which can monitor thiosulfate and drive the tunable release of the immunomodulator AvCystatin based on its fluctuations (71). By targeting specific microRNAs (miRNAs) in IBD through FMT based on the patient’s microbiome profile, these miRNAs can function in specific intestinal cells, reducing off-target effects and improving stability. This provides a new approach for developing personalized treatment strategies (72).

## 3 Gut microbiota and colorectal cancer

Chronic inflammation promotes the occurrence and development of tumors and is one of the most common risk factors for cancer. The process by which intestinal inflammation progresses to CRC through dysplasia is faster than the classic adenoma-sequence seen in sporadic CRC (73). IBD patients often exhibit the expression of inflammatory genes and excessive infiltration of inflammatory cells. This mucosal inflammation promotes cell proliferation and ultimately contributes to the development of CRC. Individuals with long-term IBD have a 2–3 times higher risk of developing CRC compared to the general population. The three most common theories regarding the development of CRC from IBD under microbial induction include the alpha-bug hypothesis, driver-passenger hypothesis, and common ground hypothesis (74). Based on genomic mutation diversity, CRC can be categorized into two main types: colitis-associated colorectal cancer (CAC) and sporadic colorectal cancer (SCC). The gut constitutes a complex environment populated by bacteria, fungi, and viruses, with an overall count potentially reaching 100 trillion. The quantity of microbial cells in the gut is projected to be around ten times that of human cells (75). Colorectal cancer can affect the makeup of the intestinal microbiota and may also be affected by the gut microbiota and their secretions. Weisburger and his colleagues released the first report associating the intestinal microbiota with CRC (76). Subsequently, an expanding body of research has confirmed the link between pathogenic bacteria and CRC.

### 3.1 Pro-carcinogenic effects of gut microbiota

Compared with healthy individuals, the gut microbiota composition of CRC individuals has experienced substantial transformations, featuring enrichment of *Firmicutes* and *Proteobacteria (* 77). Certain specific bacterial species have also been found to be related to the occurrence and development of CRC, including *Fusobacterium nucleatum*, *Escherichia coli*, *Enterococcus faecalis*, *Streptococcus gallolyticus*, and *Bacteroides fragilis (* 78, 79). These microorganisms can generate toxins and metabolites that regulate the proliferation and invasion of tumor cells, or contribute to the occurrence and progression of CRC by inhibiting immune responses, promoting the expression of oncogenes, and other means (**Table 1**).

**Table 1:** Pro-carcinogenic effects of gut microbiota.

Microbial species	Virulence factor	Mechanism	Citation
*Fusobacterium nucleatum*	FadA	Activation of the E-cadherin/β-catenin/Wnt signaling pathway	(80)
	Formate	Promote tumor stem cell renewal and activate the AhR signaling pathway	(81)
	SCFA	Regulate FFAR2-dependent Th17 response	(82)
	Hydrogen sulfide	Affect the autophagy process	(81, 83)
	Fap2	Target TIGIT to inhibit immune cell activity	(84)
	Outer Membrane Vesicle	Promote inflammatory environment by activating ERK, CREB, and NF-κB through TLR4	(85)
		Downregulate NEIL2 and induce DNA damage	(86)
		Reduce m (6)A modification through the YAP/FOXD3/METTL3/KIF26B axis	(87)
		Facilitate cell adhesion through the ALPK1/NF-κB/ICAM1 axis	(87, 88)
		Promote glycolysis by increasing lncRNA ENO1-IT1 transcription	(89)
*Colibactin-producing E.coli*	Colibactin	DNA damage	(90)
		Induce EMT and emergence of chemotherapy-resistant tumor stem cells	(91)
*Enterococcus faecalis*	O_2_^−^ and H_2_O_2_	DNA damage	(92)
	Biliverdin	Activate the PI3K/AKT/mTOR signaling pathway to promote angiogenesis	(93)
	Gelatinase	Disrupt intestinal epithelial barrier and promote inflammation	(94)
*Streptococcus gallolyticus*	Pil3 pilus	Adhere to colonic mucus	(95)
		Recruit CD11b+ TLR4+ cells to suppress immune response	(96)
		Increased expression of pro-inflammatory factors	(97)
		Activate β-catenin, c-Myc, and PCNA	(98)
		Regulate intestinal epithelial cell biotransformation pathways in an AhR-dependent manner	(99)
*Bacteroides fragilis*	BFT	Activate STAT3/Th17 immune response	(39)
	BFT	Activate NF-κB and MAPKs to induce IL-8 secretion	(100)
		Upregulate JMJD2B levels through TLR4-NFAT5 dependent pathway to regulate tumor stemness	(101)
		Downregulate miR-149-3p to promote PHF5A-mediated KAT2A RNA alternative splicing	(102)
		Regulate HDAC3/miR-139-3p pathway	(103)

#### 3.1.1 Fusobacterium nucleatum

Research shows that *Fusobacterium nucleatum* (*Fn*) is highly prevalent in CRC, with its abundance in cancerous tissues being over 400 times greater than in adjacent normal tissues (104, 105). The surface adhesion protein FadA, expressed by *Fn*, is a cell surface virulence factor and a crucial component in regulating bacterial adhesion and invasion (106). FadA attaches to cadherin on intestinal epithelial cells, activating the E-cadherin/β-catenin pathway, which affects cyclin D1, directly influencing the proliferation and growth of epithelial cells and promoting inflammatory responses and tumor formation. The FadA binding site on E-cadherin consists of an 11-amino acid stretch, and a peptide synthesized from this region can eliminate the carcinogenic effects induced by FadA. The expression levels of the FadA gene in the colon tissues of individuals with adenomas and adenocarcinomas are 10 to 100 times greater compared to those in normal individuals (80). The metabolic product of Fn, formate, has been shown to induce tumorigenesis and enhance tumor stemness, promoting glutamine metabolism and driving colorectal cancer progression through the AhR signaling pathway (81). Additionally, research has revealed that short-chain fatty acids (SCFAs) produced by *Fn* can regulate Th17 responses in an FFAR2-dependent manner, suppressing anti-tumor immune cells and facilitating tumor angiogenesis (82).

#### 3.1.2 Colibactin-producing E.coli

*Colibactin-producing E.coli* (*CoPEC*) is strongly associated with CRC (107). One study found that mucosa-associated and internalized *E. coli* levels are higher in tumors than in normal tissues, and *E.coli* colonization in the mucosa is linked to a poorer prognosis in CRC (19). *E. coli* from the B2 phylogenetic group carries a genomic island, the polyketide synthase (*pks*), which produces the genotoxin colibactin. This toxin can induce DNA damage, cell cycle arrest, mutations, and chromosomal instability in eukaryotic cells. Iftekhar et al.’s research confirmed the *in vitro* transforming potential of *pks+ E.coli*, which can undergo malignant transformation by infecting colonoid organoids, causing DNA double-strand breaks, mutations, and promoting tumor development. This emphasizes the relevance of Wnt-independent CNV mutations as early driving factors in colorectal cancer, indicating a clear connection between the mutagenic characteristics of colibactin and its action and the occurrence of colorectal cancer. Additionally, the actual transforming activity of colibactin is likely higher than the estimated activity (90).

#### 3.1.3 Enterococcus faecalis

*Efa*, similar to *E. coli*, can contribute to carcinogenesis by damaging the colonic epithelium, augmenting bacterial colonization on the mucosal surface, and facilitating epithelial cell renewal (108, 109). Huycke et al. discovered that *Efa* generates extracellular superoxide and hydrogen peroxide, leading to DNA damage. These extracellular free radicals are correlated with the advancement of adenomatous polyposis and colorectal cancer (92). Furthermore, its metabolic product biliverdin can modulate the PI3K/AKT/mTOR signaling pathway, prominently elevating the expression levels of IL-8 and VEGFA, fostering tumor cell proliferation and angiogenesis (93).

#### 3.1.4 Streptococcus gallolyticus subspecies gallolyticus

*Streptococcus gallolyticus subspecies gallolyticus* (*Sgg*), recognized as *Streptococcus bovis biotype I*, employs Pil3 pili to adhere to colonic mucus. Literature reports suggest that the abundance of *Sgg* is significantly heightened in colorectal cancer patients. *Sgg* can enlist CD11b+TLR4+ cells, promoting the expression of IL-8, COX-2, and IL-1, and escalating the levels of tumor progression-related transcription factors like β-catenin, c-Myc, and PCNA, thereby driving inflammation and facilitating tumor proliferation (95–98). It also possesses the capacity to stimulate CYP1A enzyme activity through an AhR-dependent mechanism, regulating the biotransformation pathways of colonic epithelial cells and further promoting tumor progression (99). On the contrary, the specific environmental conditions of elevated bile acid concentrations in colorectal cancer patients can facilitate *Sgg* colonization, with secondary bile acids significantly enhancing the activity of gallolysin (110).

#### 3.1.5 Bacteroides fragilis

**Figure 3:**
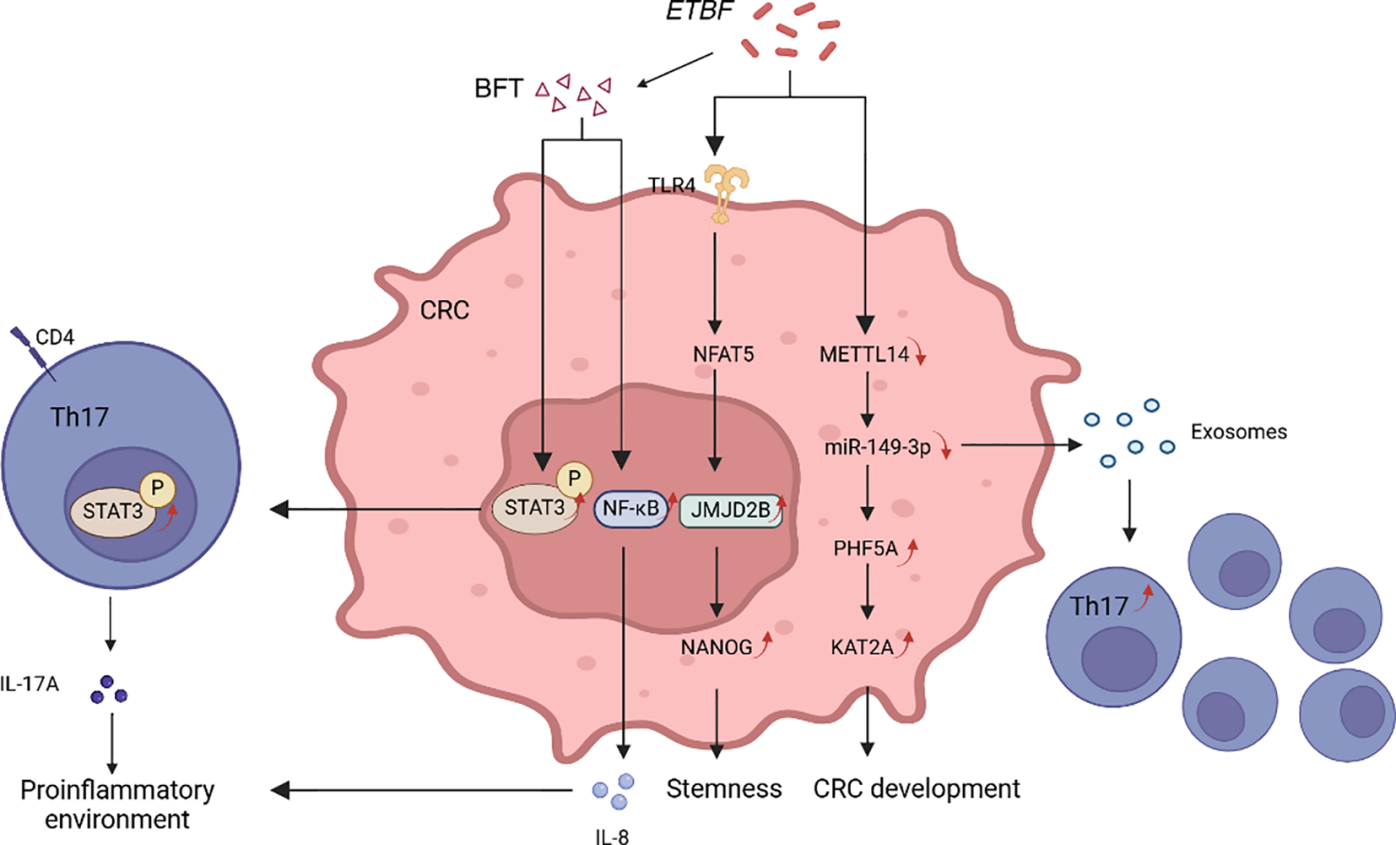
*ETBF* promotes tumorigenesis by distinct mechanisms. BFT can activate the STAT3 and NF-κB pathways, increasing the production of cytokines IL-17A and IL-8, thereby creating a pro-inflammatory environment. *ETBF* can upregulate JMJD2B levels in the TLR4-NFAT5-dependent pathway. JMJD2B regulates the stemness of tumor cells by enhancing NANOG expression through demethylation. *ETBF* can also promote cancer progression by downregulating miR-149-3p through METTL14 methylation. On one hand, it facilitates the differentiation of Th17 cells via exosomes; on the other hand, it further promotes the RNA selective splicing of KAT2A mediated by PHF5A.

*Bf* is another commensal bacterium, which is classified into toxigenic and non-toxigenic types. Research has demonstrated that the existence of *enterotoxigenic B. fragilis* (*ETBF*) is strongly linked to active IBD and CRC (**Figure 3**). The toxin BFT can stimulate c-myc expression and IL-8 release, resulting in DNA oxidation and epithelial barrier damage, and activating STAT3/Th17 immune responses (39, 111, 112). It can also activate NF-κB and upregulate COX-2 in intestinal epithelial cells, generating an inflammatory environment (100). The co-colonization of *ETBF* and enterotoxigenic *E.coli* in mice enhances the production of the inflammatory factor IL-17 and causes DNA damage, thereby accelerating the progression of CRC (113). *ETBF* can upregulate the level of JMJD2B in the TLR4-NFAT5-dependent pathway, inducing the regulation of CRC stemness (101). By reducing miR-149-3p, it additionally promotes the PHF5A-mediated RNA alternative splicing of KAT2A in CRC cells, thereby facilitating intestinal inflammation and tumors (102).

### 3.2 Anti-carcinogenic effects of gut microbiota

However, the connection between the gut microbiota and the host’s immune balance is intricate, and not all microbiota exert a cancer-promoting effect. The metabolic products of certain gut microbial groups also possess the capability to influence the anti-tumor activity of immune cells.

#### 3.2.1 Lactic acid bacteria

Certain beneficial bacteria that contribute to maintaining the ecological balance of the gut are capable of suppressing the growth of opportunistic pathogens, enhancing the barrier function of the intestinal epithelium, and typically exerting a protective role in cancer prevention. *Lactic acid bacteria*, among the most common beneficial microbes, produce organic acids that have antibacterial effects. Additionally, *Lactococcus lactis* and *Streptococcus lactis* secrete nisin, a bacteriocin that blocks the growth of most Gram-positive bacteria and their spores (114). This also implies that the role of the same bacterial group in tumors located in different parts of the digestive tract can be diametrically opposite.

#### 3.2.2 Other SCFA-producing microbiota

As one of the main metabolic products, SCFAs are essential in suppressing intestinal inflammation and serve as key protective factors in preserving the normal immune function of the intestinal mucosa, which can to some extent counteract tumor growth. Luu et al.’s research initially experimentally demonstrated that microbial metabolites like valproic acid and butyric acid boost immune cell anti-tumor activity through metabolic and epigenetic changes. Treating cytotoxic T lymphocytes (CTLs) and chimeric antigen receptor (CAR) T cells with valproic acid and butyric acid *in vitro* enhances mTOR function as a key metabolic sensor and inhibits class I histone deacetylases. This leads to anti-cancer effects and supports their use in improving cancer immunotherapy (115). Kang et al. discovered that *Roseburia intestinalis* has tumor-suppressive effects on the MSI-high and MSS subtypes of CRC. This bacterium can directly bind the butyric acid it produces to Toll-like receptor 5 (TLR5) on CD8+ T cells, activating the TLR5-dependent NF-κB pathway to enhance the function of cytotoxic CD8+ T cells, thereby exerting anti-tumor effects and enhancing the efficacy of anti-PD-1 therapy (116).

In summary, the relationship between the gut microbiota and tumors is intricate and bidirectional. Tumor progression can modify the microbiota, and alterations in the microbiota can also influence tumor progression (117). The intestinal microbiota and its metabolites significantly influence the development and progression of CRC, and clarifying the functions and potential mechanisms of the microbiota is essential for delving into more effective treatment strategies for CRC.

### 3.3 Microbiome therapy in colorectal cancer

**Figure 4:**
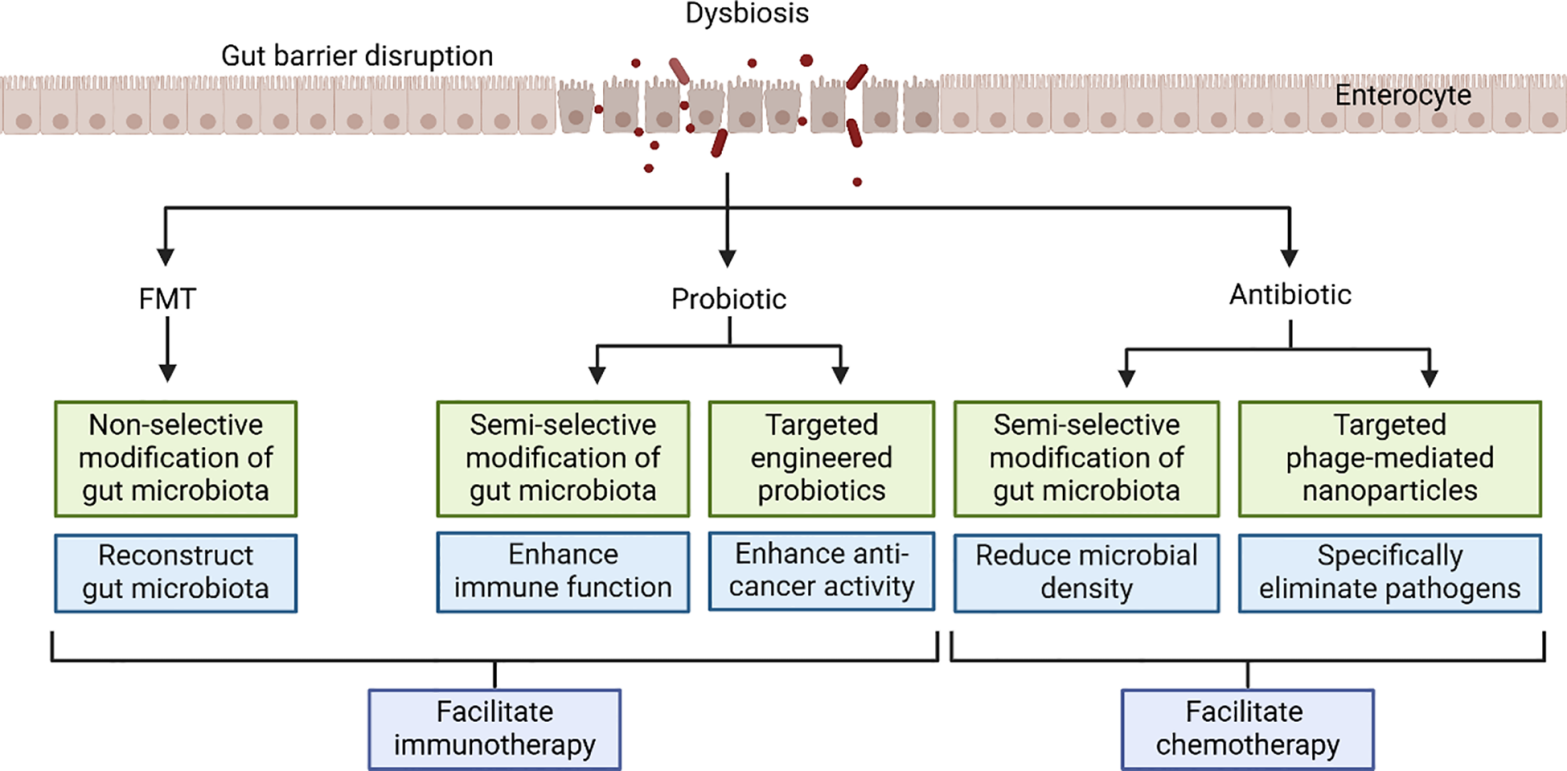
Microbiome therapies correct dysbiosis caused by colorectal cancer. Here are various methods listed from non-selective modification to targeted treatment, and they can enhance the efficacy of other colorectal tumor treatments.

Modulating the intestinal microbiota is a potential strategy for treating colorectal cancer. Various methods can alter the gut microbiota composition, improve the intestinal environment, enhance the immune system, and inhibit tumor growth (**Figure 4**).

#### 3.3.1 FMT

FMT involves introducing beneficial microorganisms obtained from the feces of healthy donors into the digestive systems of patients. This procedure aims to restore the native microbial community, increase the proportion of regulatory T cells in the mucosa, and promote cooperation among microorganisms, ultimately helping to treat both intestinal and extra-intestinal diseases (118). FMT is FDA-approved for treating refractory *Clostridium difficile* infections and has a higher cure rate compared to standard therapies (119, 120). FMT preparations can be administered orally using freeze-dried or frozen granules, or through invasive procedures like colonoscopy or gastroscopy (121). FMT has been combined with immune checkpoint inhibitors (ICIs), and a clinical trial by Routy et al. showed that FMT can boost the efficacy of anti-PD-1 therapy (122). Currently, research is predominantly centered on patients with refractory melanoma, but it indicates the potential and safety of FMT in cancer therapy. Additionally, clinical trials are ongoing to assess the utilization of FMT capsules for enhancing the efficacy of anti-PD-1 therapy in gastrointestinal cancer patients (123). The success of FMT relies on both transplanting the microbiota into the recipient’s digestive tract and ensuring long-term colonization to sustain the therapeutic benefit (124). Furthermore, FMT still bears numerous risks, with a 19% incidence of adverse events related to FMT between 2000 and 2020, typically associated with gastrointestinal discomfort, and severe adverse events mainly occur in patients with mucosal barrier damage (125). Clinical research on FMT as an adjuvant therapy for cancer is limited; thus, it is of paramount importance to standardize its application to ensure its safety and long-term efficacy.

#### 3.3.2 Probiotic

Probiotics, being beneficial microorganisms for the host’s health, can inhibit tumor cell proliferation by regulating the gut microbiota and immune response (126, 127). Compared with FMT, they notably reduce the risk of infection for patients. Commonly utilized probiotics such as *Bifidobacterium* and *Lactobacillus* have recognized effects in treating various gastrointestinal disorders (128). Research has indicated that *Lactobacillus plantarum* can suppress the proliferation of colon cancer cells through the cell cycle regulatory ability mediated by butyric acid (129). Many studies regarding the application of probiotics to treat cancer focus on enhancing immune function, which might assist in combating cancer (120). For instance, Bifidobacterium plays a role in strengthening anti-tumor immunity in anti-PD-L1 treatment (130). There are also investigations on engineered probiotics for targeted tumor therapy (131). The substrate of probiotics—prebiotics, has also been demonstrated to participate in tumor treatment. Fructo-oligosaccharides (FOS) and galacto-oligosaccharides (GOS) are two significant groups of prebiotics that selectively encourage the proliferation of beneficial probiotics. They increase the production of SCFAs, which lead to subtle modifications in the intestinal microbiota and potentially enhance the effectiveness of oncology treatments (132–134). Probiotics and prebiotics hold great potential in the domain of cancer treatment.

#### 3.3.3 Antibiotic

Besides augmenting the profusion of probiotics, the application of antibiotics for eradicating pathogenic microorganisms constitutes another strategy for manipulating the gut microbiota. Clinical trials have emerged demonstrating the preventive utilization of rifaximin to curtail infections, gastrointestinal toxicity, and diarrhea associated with cancer treatment (135). Rifaximin proves efficacious in preventing recurrent/refractory *Clostridium difficile* infections, regulating bacterial metabolism, and enhancing mucosal barrier function. Preventive antibiotic treatment is frequently employed in combination with chemotherapy or immunotherapy; however, studies have discovered that antibiotic administration is correlated with diminished chemotherapy efficacy, decreased responsiveness to immune checkpoint inhibitors (ICIs), and adverse prognosis (136, 137). It can curtail the effectiveness of PD-1 blockade in cancer individuals via the MAdCAM-1-a4b7 axis, adversely influencing prognosis (138). Antibiotics can remove pathogenic microorganisms, though they may also disturb the balance and composition of the body’s microbial community, detrimental to beneficial bacteria, and giving rise to dysregulation of host-microbiota interactions (139). They can reduce the gut microbial load by a factor of 10,000, and some specific microbial species could remain absent for an extended duration after their administration (5). With the progression of nanotechnology, the targeted delivery of antibiotics for selectively eliminating pathogenic microorganisms provides more potentialities for adjusting the gut microbiota to treat digestive disorders. Nanomaterials, serving as carriers, convey therapeutic drugs to target sites, protecting them from degradation and reducing their accumulation in non-target areas, which helps to minimize side effects (140, 141). The targeted action of bacteriophages holds significance for the specific elimination of pathogenic microorganisms (142). Research has devised bacteriophage-mediated nanoparticles targeting nuclear-positive *fusobacteria*, augmenting the chemotherapeutic effect on CRC (143). Nanomedicine might be an efficacious treatment strategy for regulating the intestinal microbiota in the future.

#### 3.3.4 Diagnostics and personalized medicine

The gut microbiota exhibits unique variability and plasticity among different individuals, making it an important component of personalized medicine. Some studies have already utilized gut microbiota data by integrating multi-omics data to detect CRC. Mulenga et al. proposed a novel feature engineering method that can accurately classify CRC using gut microbiota data through a deep neural network (DNN) model (144). Additionally, research has shown that Raman spectroscopy (RS) can enable real-time analysis of microbial community composition and metabolic activity, further achieving the goals of discovering biomarkers, enhancing diagnostic potential, and enabling personalized treatment (145). Currently, engineered microorganisms are receiving more attention due to their potential applications in the diagnosis and treatment of CRC and IBD (146). A study utilized genetic engineering to enable engineered commensal E. coli to specifically adhere to the surface of CRC cells. By secreting myrosinase, these bacteria convert natural compounds from cruciferous vegetables consumed by the host into organic small molecules with anti-cancer activity, significantly inhibiting tumor proliferation (131). Furthermore, combining gut microbiota with precision medicine is an important approach to enhance drug efficacy and reduce drug toxicity (147). Linking microbiota research with clinical diagnostic and therapeutic strategies holds promise for achieving microbiota-based diagnostics and personalized medicine.

### 3.4 Gut microbiota modulates other colorectal tumor therapies

Recently, it has been discovered that the intestinal microbiome is strongly associated with the efficacy, toxicity, and side effects of commonly used colorectal cancer treatments. The gut microbiome has different regulatory effects on chemotherapy and immunotherapy; it can improve the effectiveness of immunotherapy while promoting resistance to chemotherapy.

#### 3.4.1 Gut microbiota promotes chemotherapy resistance

The intestinal microbiota can modulate chemotherapeutic drug metabolism, affecting cancer response to treatment and the host’s sensitivity to toxicity (148). Geller et al. are of the opinion that bacteria can metabolize the chemotherapeutic drug gemcitabine into its inactive form, giving rise to gemcitabine resistance. This metabolic process hinges on the expression of a long isoform of the bacterial enzyme cytidine deaminase (CDDL), which is predominantly present in *Gammaproteobacteria*, and co-treatment with the antibiotic ciprofloxacin can eliminate this resistance (149). The gut microbiota also influences the anticancer activity of cyclophosphamide, cisplatin, and 5-FU (150–152). These effects might be associated with the translocation of Gram-positive bacteria in the course of mucosal inflammation, subsequently triggering cytotoxic ROS and causing pathogenic Th17 cells to invade tumors (148, 151). Translocated bacteria can interact with the immune system and induce inflammation, thereby affecting the efficacy of chemotherapy (153). Cancer treatment demands an intact commensal microbiota to attain the optimal response, which mediates its effects by regulating the function of myeloid-derived cells in the tumor microenvironment (150). Metabolites of the gut microbiota can also modulate the efficacy of chemotherapy. For example, 3-Oxocholic acid, a metabolite associated with *Prevotella*, reduces the chemotherapeutic effect of FOLFOX *in vitro (* 154). Additionally, after *CoPEC* infects CRC cells, the production of colibactin induces the accumulation of lipid droplets in cancer cells, limiting genotoxic stress to some extent. CoPEC infection also enhances phosphatidylcholine remodeling through enzymes in the Land’s cycle, providing cancer cells with sufficient energy to sustain survival during chemotherapy, thereby leading to chemoresistance (155). The intestinal microbiota is closely linked to the effectiveness of tumor chemotherapy, but more research is needed to elucidate the mechanisms by which these differential or chemotherapy-adapted bacteria influence chemotherapy responses.

#### 3.4.2 Gut microbiota facilitates tumor immunotherapy

**Figure 5:**
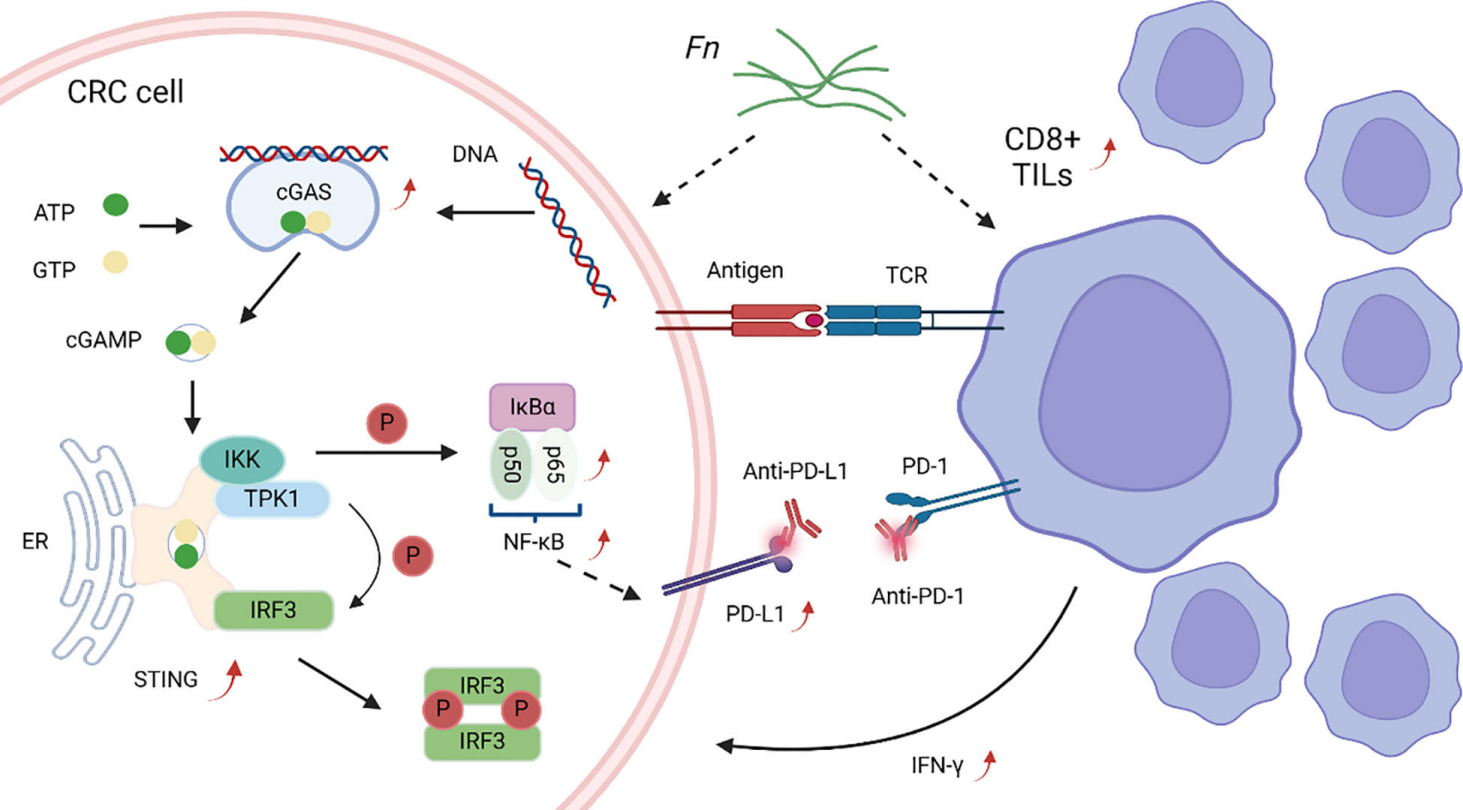
*Fn* involved in immunotherapy. *Fn* can activate the cGAS-STING signaling pathway in CRC cells, thereby upregulating PD-L1 expression through NF-κB (p65) transcription and enhancing anti-tumor effects. *Fn* can also recruit IFN-γ+ CD8+ TILs, increasing IFN-γ production and enhancing the efficacy of anti-PD-L1 therapy, thereby killing tumor cells.

The objective of immune checkpoint blockade is to restore and augment the capacity to assail cancer cells by suppressing the tumor’s immune resistance. In immune therapy-related investigations, the two most targeted immune checkpoint regulatory factors are cytotoxic T lymphocyte-associated antigen-4 (CTLA-4) and programmed cell death protein-1 (PD-1) or its ligand PD-1 ligand-1 (PD-L1). Presently, immune checkpoint inhibitors (ICI) have been sanctioned by the FDA for the treatment of specific CRC. The gut microbiota has been shown to affect how cancer responds to checkpoint inhibitors. Sivan et al. found that the gut microbiota can influence the effectiveness of anti-PD-1/PD-L1 monoclonal antibodies in mice, with *Bifidobacterium* being correlated with anti-tumor effects by activating dendritic cells, leading to the activation and infiltration of CD8+ T cells in the tumor microenvironment to enhance the efficacy of immunotherapy (130). Oral administration of *Bifidobacterium* alone yielded anti-cancer effects similar to PD-L1-specific antibody therapy, and the combination of the two nearly completely inhibited tumor growth. Manipulating the microbiota might regulate cancer immunotherapy. Research indicates that *Fn* may counteract CRC by altering the tumor immune microenvironment. Gao et al. reported that *Fn* stimulates PD-L1 expression via the STING signaling pathway and boosts the presence of IFN-γ (+) CD8 (+) tumor-infiltrating lymphocytes (TILs), thereby enhancing the tumor’s sensitivity to PD-L1 blockade and exerting anti-tumor effects(**Figure 5**) (156). In certain cases, the gut microbiome is closely related to the response to ICI and may enhance the anti-cancer effects induced by immunotherapy, including immune responses at the tumor site, although the exact mechanisms remain unclear (157). Recently, Wang et al. elucidated that *Fn* and its abundant butyrate production can increase the sensitivity of microsatellite stable (MSS) CRC to PD-1 treatment, thereby improving treatment outcomes (158). Additionally, the utilization of antibodies may disrupt the equilibrium of immune tolerance, resulting in the development of autoimmune diseases. Research has shown that *Bifidobacterium* can alleviate CTLA-4 blockade-induced intestinal mucosal immune reactions without significantly influencing anti-tumor immunity (159). Lo et al. found that colitis induced by anti-CTLA-4 therapy is related to the composition of the gut microbiota, and the inflammation is caused by the unchecked activation of CD4+ T cells and the attenuation of Tregs through their interaction with the Fc domain of the CTLA-4 antibody (160). In summary, the intestinal microbiota can impact the clinical prognosis of cancer immunotherapy.

## 4 Clinical relevant research

Modulating the gut microbiota can play an important role in the treatment of IBD and CRC, and some progress has been made, but extensive research is still needed to determine the efficacy of these treatment options. Several clinical trials have reported that FMT, probiotics, and prebiotics can manipulate the gut microbiota to treat IBD (**Table 2**) (15). Although the safety and long-term efficacy of FMT require further evaluation, the majority of studies suggest that it has a beneficial impact on achieving clinical, endoscopic, and histological remission (161). VSL#3 has been confirmed by multiple clinical studies to effectively improve IBD with a certain degree of safety. It can reduce endoscopic recurrence after CD surgery, induce remission in patients with mild to moderate active UC, and alleviate inflammation. Its combination with standard therapy is more effective in treating UC than standard therapy alone (15, 162). EcN has also been recognized through double-blind trials as an alternative to mesalazine for the treatment of UC, demonstrating efficacy and safety comparable to the gold standard in maintaining remission (163).

**Table 2:** Summary of key clinical trials.

Clinical Trial ID	Study title	Patient population	Intervention	Main finding
NCT00175292	A randomized controlled trial of VSL#3 for the prevention of endoscopic recurrence following surgery for Crohn’s disease	CD	VSL#3	VSL#3 can prevent serious endoscopic recurrence.
NCT00114465	VSL#3 versus placebo in maintenance of remission in Crohn’s disease	CD	VSL#3	VSL#3 demonstrates efficacy in preventing pouchitis onset.
NCT00803829	Synbiotic treatment of ulcerative colitis patients	UC	Synbiotic (Synergy 1/*B. longum*)	Short-term treatment improved the clinical manifestations of chronic inflammation.
NCT04102852	*Lactobacillus rhamnosus* GG (ATCC 53103) in mild-moderately active ulcerative colitis patients	UC	*Lactobacillus rhamnosus* GG (LGG)	LGG effectively exerts anti-inflammatory effects.
NCT04969679	Additive effect of probiotics (Mutaflor^®^) in patients with ulcerative colitis on 5-ASA treatment	UC	*E. coli* Nissle 1917 (Mutaflor^®^)	EcN can prevent disease progression in mild-to-moderate patients and achieve both clinical and endoscopic remission.
NCT01896635	Fecal microbiota transplantation in ulcerative colitis (FOCUS)	UC	FMT infusions	Intensive dosing and multi-donor FMT can induce clinical remission and endoscopic improvement.
NCT02460705	Fecal microbiota transplant for inflammatory bowel disease	CD	Biologically active human fecal material (OpenBiome)	Single-dose FMT demonstrates modest therapeutic efficacy but is associated with potential risks.
NCT01560819	Gut microbial transplantation in pediatric inflammatory bowel diseases (GMT)	UC	FMT	FMT contributes to clinical remission with acceptable adverse effects.
NCT01847170	Impact of fecal biotherapy (FBT) on microbial diversity in patients with moderate to severe inflammatory bowel disease	CD	FMT	FMT increases intestinal microbial diversity with an acceptable safety profile.
NCT02097797	Impact of the fecal flora transplantation on Crohn’s disease (IMPACT-Crohn)	CD	FMT	Higher colonization by donor microbiota was associated with maintenance of remission.
NCT02516384	Fecal microbiota transplantation (FMT) in the management of ulcerative colitis (UC)	UC	FMT	Odoribacter splanchnicus in FMT recipients limits colonic inflammation.
NCT01545908	Fecal biotherapy for the induction of remission in active ulcerative colitis	UC	Fecal microbiota enema	FMT induced clinical remission in patients, with no significant difference in adverse events compared to the control group.

Additionally, there is additional data on the utilization of gut microbiota to assist in the treatment of CRC. For patients undergoing chemotherapy after CRC surgery, the intake of probiotics can effectively mitigate chemotherapy-induced disruptions in gut microbiota and gastrointestinal complications such as diarrhea (164). Moreover, probiotic therapy is beneficial for postoperative recovery, associated with lower individual postoperative mortality rates, shorter hospital stays, and a reduction in surgical site infections and postoperative ileus, among other complications (165, 166). In summary, the application of probiotics can enhance the efficacy of anti-cancer treatments, reduce CRC complications, and improve prognosis. While numerous *in vitro* and animal experiments have demonstrated the potential of gut microbiota in adjuvant CRC treatment, clinical research remains limited, particularly trials related to FMT. Issues such as how to alleviate adverse reactions when used in conjunction with other anti-cancer drugs still need to be addressed.

## 5 Conclusion

The composition and quantity of intestinal microbiota vary in different states of the human body. The diversity of *Firmicutes* exhibits a decreasing tendency in IBD patients but significantly rises in CRC patients. The same microbial community can play diverse roles in different circumstances. Although *LAB* is a key metabolite in the carcinogenic process of GC, facilitating the colonization of related pathogens in the stomach, while also exerting antibacterial and bactericidal effects in the intestine, playing a protective role in preventing CRC. *Fn*, a prevalent anaerobic bacterium found in the oral cavity, is an opportunistic pathogen closely related to CRC. Its cell surface virulence factors and metabolic products can promote tumor formation and progression, while also enhancing the sensitivity of tumors to immunotherapy and inducing effective anti-cancer effects. The article also mentions that *Efa* and *ETBF* both can boost inflammatory responses in IBD and have cancer-promoting effects on CRC. Nevertheless, *NTBF* and *Bf HCK-B3* have positive impacts on colonic epithelial cells, enhancing barrier function, alleviating inflammation, and promoting immune tolerance.

The heterogeneity of the digestive tract microbiota offers a strong impetus for personalized treatment strategies for IBD and tumors due to its high efficacy and targeted nature. With the progress in detection and identification technologies, the development of targeted therapies based on individual microbiota, such as antibodies or other biological agents, has become more feasible. Currently, common probiotics such as *Bifidobacterium* and *Lactobacillus* can be used alongside other anti-tumor treatments, like immunotherapy and chemotherapy, to achieve better synergistic effects. Engineered bacteria and FMT treatments are also being explored, aiming to improve IBD inflammation or normalize tumor immunity by regulating the intestinal microbiota. However, several challenges remain unresolved, such as the lack of standardization in FMT preparation and delivery protocols.

Future research must first further clarify the specific mechanisms by which gut microbiota influence IBD and tumors, particularly how different microbial communities and their metabolites affect host immune responses and the tumor microenvironment. Additionally, the synergistic mechanisms between microbiota and other anti-cancer therapies also require in-depth exploration. Furthermore, how to achieve personalized diagnosis and treatment through precise regulation of microbiota is a critical issue, including the development of engineered bacteria and the optimization of FMT technology. Finally, reducing the risks associated with microbiota-related treatments and improving their safety and long-term efficacy are also key points for future research. The gut microbiota exhibits a “double-edged sword” characteristic in both IBD and CRC, necessitating therapeutic strategies that carefully balance treatment efficacy with safety considerations. With technological advancements and deeper investigations, microbiota-related research is expected to open new avenues for the treatment of IBD and tumors.
